# A novel and rapid method to purify the human complement opsonin C3b from human plasma

**DOI:** 10.3389/fimmu.2025.1639735

**Published:** 2025-08-26

**Authors:** Jannik Sichau, Christoph Q. Schmidt

**Affiliations:** Institute of Experimental and Clinical Pharmacology, Toxicology and Pharmacology of Natural Products, University of Ulm Medical Centre, Ulm, Germany

**Keywords:** complement - immunological terms, opsonins, C3b, purification method, factor I

## Abstract

Complement opsonin C3b is at the center of the complement cascade. All complement initiation pathways converge when convertases activate C3, converting it into the anaphylatoxin C3a and the opsonin C3b, which can attach to surfaces via its freshly exposed thioester moiety. C3b has several important functions and remains a focus of basic and translational research. C3b and its proteolytically processed derivatives iC3b and C3dg are important opsonins for different complement receptors on various immune cells. C3b is the initiation nucleus for the formation of the alternative pathway C3 convertase and C3b clusters act as gatekeepers for the activation of C5, which initiates the terminal pathway. High densities of surface-deposited C3b alter the substrate specificity of complement convertases from C3 to C5 activation. It remains unknown how C3b achieves the “C5 priming” necessary for this process. Further exploration of the functions of C3b is laborious, as it generally requires the purification of C3 from plasma/serum, subsequent conversion to C3b, and further purification. Therefore, we developed a rapid (three days) and efficient method that purifies C3b directly from blood sources, requiring fewer steps and reagents than previously published protocols. This method utilizes the newly exposed sulfhydryl group, which becomes accessible by converting C3 to C3b. The C3b purified by this method exhibits high purity and very high activity, as demonstrated by affinity measurements for several known C3b ligands, the formation and dissociation of the alternative pathway convertase, and the capacity to be regulated by FI.

## Introduction

With more than ten clinically approved complement inhibitors, the complement field has definitely proven to be suitable for therapeutic intervention (1). Despite this remarkable progress, several challenges remain. For several diseases with a clear complement-driven pathology, inhibitors are not yet available for clinical use. Several of the approved inhibitors exhibit unexpected behavior during treatment. Examples include extravascular haemolysis of PNH erythrocytes or pharmacodynamic breakthrough leading to intravascular haemolysis during anti-C5 treatment (2–6), reviewed in (7). Recently, breakthrough haemolysis has also been described for the C3 inhibitor pegcetacoplan in the treatment of PNH patients (8–11). However, unexpected haemolysis events are not unique to C3 and C5 inhibition. Intravascular haemolysis has also occurred with the clinical use of other complement inhibitors targeting the proximal complement pathway (12, 13). This calls for further research to answer urgent fundamental questions about the complement pathways associated with these unexpected clinical findings when complement therapeutics are used in the clinic. Many questions that remain to be answered center around the complement opsonin C3b. Arguably, the most important question is how the clustering of C3b molecules prepares or primes C5 for proteolytic cleavage by complement convertases. It is expected that addressing this question experimentally will require milligram amounts of C3b (5, 14–17).

Although C3b is commercially available as a plasma/serum-purified protein, experiments requiring large quantities are relatively expensive. As an alternative, several published purification protocols detail how to obtain C3b at various laboratory scales. **Table 1** summarizes selected published protocols for obtaining C3 and/or C3b (18–25). All available C3b purification protocols first purify C3 from plasma or serum, then convert C3 to C3b using specialized techniques such as limited trypsin digestion or processing by cobra venom factor (CVF) convertases. This process requires CVF and purified Factor B and Factor D. Purifying functional C3 from blood sources requires five to eight steps. However, when C3 is only purified as an intermediate to be further processed into C3b, four steps are sufficient (**Table 1**). Although these C3 preparations are expected to still contain impurities, it is assumed that their level of purity is sufficient for subsequent conversion to C3b, followed by further purification steps that yield pure C3b preparations. One protocol for small-scale purification involves a total of seven steps (four steps to obtain the intermediate C3 and three to obtain C3b). Another protocol involves nine steps to obtain C3b (four steps to isolate C3, followed by five steps to generate and purify C3b from C3) for larger-scale purification.

**Table 1:** Summary of selected C3 (red) and C3b (blue) purification protocols. Further necessary steps to convert C3 to obtain C3b (pink).

Method step	Nilsson et al., 1965 (18)	Hammer et al., 1981 (19)	Scott et al., 1982 (20)	Rear et al., 1994 (21) [preparative FPLC chromatography]	Van den Berg, 2000 (22)	Van den Berg, 2000 (22)	Janssen et al., 2006 (24) & Lambris et al., 1980 (25): combined protocol	New method
#0	Serum preparation (large* scale)	Plasma preparation (large* scale)	Serum preparation (*intermediate/large scale*)	Plasma preparation (large* scale)	Plasma preparation (large* scale)	Serum preparation *(small scale ≤5 ml serum)*	Plasma preparation *(intermediate/large scale)*	Plasma preparation *(intermediate scale)*
#1	Buffer exchange to pH 5.4 (dialysis, leading to precipitation) & re-suspension at pH 8.1	5% PEG4000 precipitation (supernatant was used)	5% PEG4000 precipitation (supernatant was used)	Plasminogen depletion by lysine agarose (affinity chromatography)	5% PEG4000 precipitation (supernatant was used)	4% PEG6000 precipitation (supernatant was used)	Plasminogen depletion by lysine agarose (affinity chromatography)	Factor I depletion with anti-FI beads**
#2	Euglobulin precipitation and lipid depletion by centrifugation	Plasminogen depletion with Sepharose 4B-L-lysine (affinity chromatography)	12% PEG4000 precipiation (precipitate was used)	Buffer exchange (concentrator)	12% PEG4000 precipitation (precipitate was used)	10% PEG6000 precipitation (precipitate was used)	5% PEG4000 precipitation (supernatant was used)	Blocking of free thiol-groups with iodacetamide
#3	Buffer exchange (dialysis) for AEX	Buffer exchange (concentrator) for AEX	Alternative Pathway activation (37°C incubation)	5% PEG4000 precipitation (supernatant was used)	Plasminogen depletion with Sepharose 4B-L-lysine	MonoQ column (AEX) at pH 8.9	Buffer exchange (concentrator) for AEX	Buffer exchange (dialysis) to deplete iodacetamide
#4	TEAE column (AEX) at pH 8.1	DEAE Sephacel column (AEX) at pH 7.4	DEAE Sepharose CL-6B column (AEX)	Buffer exchange (concentrator) for AEX	Dilution with AEX buffer and purification with DEAE column (AEX) at pH 7	Superose 12 FPLC column (SEC)	DEAE Sephacel column (AEX) at pH 7.8	Activated-Thiol-Sepharose (ATS) bead purification
#5	Concentrating by precipitation at pH 5.4	16% PEG4000 precipitation (precipitate was used)	Buffer exchange (dialysis)	DEAE Sephacel column (AEX) at pH 8	16% PEG4000 precipitation (precipitate was used)	Conversion of C3 to C3b with CVF-Sepharose** (pre-incubated with serum)	Conversion of C3 to C3b with 1% trypsin/substrate ratio**	MonoQ column (AEX) at pH 7.4
#6	Hydroxyl-apatite column (HAC)	Sepharose CL-6B column (SEC)	FH-Sepharose 4B column (affinity chromatography)**	16% PEG4000 precipitation (precipitate was used)	Buffer exchange (dialysis) for HAC purification	(MonoQ column (AEX) to separate remaining C3 from C3b at pH 8.9)	Superdex 200 pg column (SEC) at pH 7.4	Superdex 200 pg column (SEC) at pH 7.4
#7	Buffer exchange to pH 5.4 (dialysis, leading to precipitation) and re-dissolving at pH 7	Traces of C5, IgG and IgA were removed by immune adsorption with Sepharose 4B anti-C5, -IgG and -IgA	Buffer exchange (dialysis) into 1% (w/v) NH_4_HCO_3_	S-300 HR column (SEC)	Hydroxyl-apatite column (HAC) at pH 7.4	Sephadex G-100 column (SEC)	MonoQ column (AEX) at pH 7.5	
#8	Additional steps for conversion to C3b by any method**	Additional steps for conversion to C3b by any method**		[Dialysis and MonoQ (AEX) at pH 7.3]	Additional steps for conversion to C3b by any method**		MonoS column (CEX) at pH 6	
#9				[Depletion of C3(H2O) or inactivated C3 with ATS beads]			Buffer exchange and concentrating at pH 7.4 (concentrator)	
#10				Additional steps for conversion to C3b by any method**				

*large scale means > 100 ml; ** requires purified protein as a reagent within the purification process.

Here, we describe a novel protocol for purifying human C3b directly from plasma eliminating the need for intermediate C3 purification. Consisting of only six steps, this protocol is at least as efficient as the previously published methods, avoiding protein precipitation steps and the use of convertase reagents such as Factor B and Factor D to convert C3 to C3b. This is achieved by selectively depleting the complement-specific serum protease, Factor I (FI), prior to complement activation in plasma, and utilizing the released sulfhydryl group when C3 is converted to C3b. To the best of our knowledge, this is the first protocol to describe the direct purification of C3b from a blood source without any intermediate C3 purification.

## Materials and methods

### Protein reagents

C3b, Factor D (FD), Factor B (FB) and FI (FI) were purchased from Complement Technology. The recombinant proteins, which include complement receptor 1 (CR1) spanning the complement control protein (CCP) domains 1-3 [*i.e*. CR1(1-3)], CCPs 15-17 [*i.e.* CR1(15-17)], complement Factor H CCPs 1-6 [*i.e.* FH(1-6)], CCPs 19-20 [*i.e.* FH(19-20)] and the variable domain of the complement receptor of the immunoglobulin superfamily (*i.e.* CRIg), were produced as previously described (17, 26–28). Briefly, the proteins were expressed in the heterologous host *Pichia pastoris* and purified to homogeneity using a series of conservative purification steps involving a combination of ion-exchange and size-exclusion chromatography steps.

### Generation of polyclonal anti-FI resins

#### Generation of resins for affinity chromatography

CNBr-activated Sepharose 4B beads (Cytiva) were prepared according to the manufacturer’s instructions. In brief, the lyophilized beads were resuspended and washed with 1 mM HCl on a sintered glass filter for 15 min. The resin was washed with 100 mM sodium carbonate buffer containing 500 mM NaCl at pH 8.3 in a gravity-flow column (BioRad). Then, the ligand was added. The beads were then incubated with the ligand at ambient temperature for 1 h while rotating on a rotator. The supernatant was discarded, and the beads were blocked with 100 mM Tris-HCl at pH 8 at ambient temperature for 2 h while rotating. Then the beads were washed with at least five column volumes (CV) of 100 mM sodium acetate containing 500 mM NaCl at pH 4, then with 100 mM Tris-HCl containing 500 mM NaCl at pH 8, and finally with PBS. These three consecutive washing steps were performed a total of three times. The following amounts of protein were coupled to the beads: Affinity-purified FI: 4 mg of protein on 1.5 ml of beads. Polyclonal sheep anti-human FI antibody: 12 mg of affinity-purified anti-FI pAb on 4 ml of beads. The protein coupled to the beads is stored in PBS with 20% ethanol at 4°C and can be reused.

#### FI and polyclonal anti-FI antibody purification

FI was obtained by an in-house affinity-purification from plasma/serum using polyclonal anti-FI antibodies coupled to sepharose beads. In brief, the beads were added to human plasma and incubated at 4°C for 2 h on a tilting table. The plasma was collected from the beads using a gravity flow column (BioRad). Then, the beads were washed with 30 CV of PBS, 40 CV of PBS containing 500 mM NaCl and finally 20 CV of PBS. The FI was eluted by the stepwise addition of 1 CV of 100 mM glycine buffer at pH 2.2 until no further protein elution was detected (by absorbance at 280 nm). The elution steps were collected in tubes containing 0.2 CV of 1 M Tris-HCl at pH 8. Then the beads were washed with 80 CV of PBS to prepare them for another cycle of FI capture from fresh plasma. This cycle was repeated until no more FI protein eluted from the beads. The eluted protein was concentrated and then the buffer was exchanged for PBS using an Amicon Ultra centrifugal filter with a 3 kDa cut-off (Merck). FI was further purified by size-exclusion chromatography using a HiLoad 16/600 Superdex 200 pg column (Cytiva), and the buffer was exchanged for a 100 mM sodium carbonate buffer containing 500 mM NaCl at pH 8.3.

The polyclonal anti-FI antibody was purified from anti-human FI serum obtained from goats or sheep (Quidel/Complement Technology/Eurogentec, a service provider for polyclonal antibodies) by affinity chromatography. The anti-FI serum was added to FI-coupled Sepharose beads and incubated at ambient temperature. Elution was performed using a 100 mM glycine buffer at pH 2.0. The eluted protein was concentrated, and the buffer was exchanged for 100 mM sodium carbonate buffer containing 500 mM NaCl at pH 8.3 using an Amicon Ultra centrifugal filter with a 3 kDa cut-off (Merck).

### C3b purification

#### Plasma collection

Fresh blood was collected from three healthy donors into three 9 ml VACUETTE/Monovette EDTA (1.6 mg/ml) - blood collection tubes. The tubes were then centrifuged at 2600 x g for 15 minutes at 4°C. The plasma was separated and stored on ice.

#### FI – depletion

The FI affinity resin (polyclonal anti-FI antibodies coupled to Sepharose) was washed with 30 CV of PBS. The plasma was added to the resin in a gravity-flow column and incubated at 4°C for 2 h on a tilting table or rotator. Then, the plasma was allowed to pass through the resin, was collected and kept on ice. The resin was washed with 15 CV of PBS, followed by 20 CV of PBS containing 500 mM NaCl, and a final wash with 10 CV of PBS. During these washing steps, the flow rate was increased by applying air pressure. FI was eluted by stepwise addition of 1 CV of 100 mM glycine buffer at pH 2.2 and collected into tubes containing 0.2 CV of 1 M Tris-HCl at pH 8. Elution was continued until no protein could be detected by absorbance measurements at 280 nm. The resin was re-equilibrated with 30 CV of PBS. Then, the plasma that has passed through the affinity resin was applied for another cycle. Capture, elution and equilibration were repeated as described above.

#### Blocking of free thiol groups and dialysis

The FI-depleted plasma was supplemented with 20 mM iodoacetamide. The suspension was incubated at ambient temperature for 1 h on a rotator. The plasma was transferred to a SnakeSkin dialysis tube with a 10 kDa cut-off and placed in a container filled with 2 l of PBS supplemented with 5 mM EDTA. Dialysis proceeded at 8°C with gentle stirring for 2 h. A total of three dialysis procedures were performed with fresh dialysis buffer, involving two 2 h incubations and one overnight incubation.

#### Generation, capturing, and elution of C3b

1.3 g of activated thiol Sepharose (ATS) 4B beads (Cytiva) were added to a gravity flow column, resuspended in water, and incubated for 10 min. The beads were washed with 10 CV of water and 10 CV of PBS. The dialyzed plasma (from the step above) was transferred to the ATS beads. EGTA and MgCl_2_ were added to achieve a final concentration of 10 mM each. The mixture was incubated at 37°C for 2 h on a rotator. The ATS beads were washed with 15 CV of PBS, followed by 20 CV of PBS containing 500 mM NaCl, and finally 10 CV of PBS. The protein was eluted by the addition of 2 CV of freshly prepared 1 mM cysteine dissolved in PBS and incubation at 30°C for 1 h while rotating at 35 rpm. Then, the eluted protein was collected by gravity flow into a single container. To wash the beads and dilute the cysteine content in the elution sample, the resin was washed with 8 CV of 20 mM sodium phosphate buffer containing 5 mM EDTA at pH 7.4 and collected into the same container. The protein was stored on ice until further use. This elution/wash/dilution step was repeated five times in total. The protein quality of each consecutive elution step was assessed by SDS-PAGE.

#### Further polishing

The fraction of each elution cycle was pooled and applied to an anion-exchange-chromatography column (MonoQ 10/100 GL, Cytiva). The column was washed with 20 mM sodium phosphate buffer containing 5 mM EDTA at pH 7.4. Elution was achieved by gradually increasing the salt concentration to 1 M over 20 CV. The fractions were analyzed by SDS-PAGE. C3b-containg fractions were collected and concentrated using an Amicon Ultra centrifugal filter with a 3 kDa cut-off (Merck). The concentrated protein was applied to a size-exclusion column (HiLoad 16/600 Superdex 200 pg, Cytiva) and concentrated before being analyzed directly or frozen in liquid nitrogen for storage at -80°C. Chromatograms were recorded using the ChromLab software (Bio-Rad) and depicted using OriginPro^®^ evaluation software. The concentration of purified C3b was determined by measuring the absorbance at 280 nm and using the same extinction coefficient provided by Complement Technology, i.e. 1 mg/ml C3b results in an absorbance at 280 nm of 1.03 with the molecular weight being 176 kDa.

### Biotinylation of C3b

To ensure the reduced state of the sulfhydryl groups, C3b was incubated in PBS with 1 mM DTT at ambient temperature for 2 h. The buffer was then exchanged for PBS using an Amicon Ultra centrifugal filter with a 10 kDa cut-off (Merck). Biotinylation was performed using the EZ-Link maleimide-PEG_2_-biotin kit (Thermo Fisher Scientific) according to the manufacturer’s protocol with a molar ratio of 1:20 of C3b to maleimide-biotin. The buffer was exchanged for PBS as before.

### Surface plasmon resonance experiments

All SPR measurements were performed at 25°C using a Biacore X100 instrument (Cytiva). PBS supplemented with 0.05% Tween20 and 1 mM MgCl_2_ was used as the running buffer. The chip surface was conditioned (according to the manufacturer’s protocol) three consecutive times by injecting 50 mM NaOH containing 1 M NaCl at a flow rate of 30 µl/min for 1 min. After each injection, the needle was washed with 50 mM NaOH with 1 M NaCl and 50% isopropanol. Biotinylated C3b was injected onto a SA sensor chip (Cytiva) at a flow rate of 10 µl/min until an immobilization level of 950–1050 RU was reached.

For the affinity measurement, analytes were injected at a flow rate of 30 µl/min for 2 min, after which the surface was regenerated by injecting 1 M NaCl for 30 s. A 1:1 dilution series of CRIg, CR1(15-17) and FH(19-20) was tested with the highest concentrations being 10 µM, 20 µM and 40 µM, respectively. The highest concentration of each analyte was injected three times: at the first and the last two cycles. A series of increasing concentrations was injected in between. The response levels at the end of the injection were used to determine the equilibrium dissociation constant using a 1:1 steady-state binding model in the Biacore X100 evaluation software (Cytiva). The data were exported and depicted in the OriginPro^®^ evaluation software.

For convertase formation, a mixture of 600 nM FB and 100 nM FD was injected at a flow rate of 30 µl/min for 3 min, followed by a 10 min dissociation period. Regeneration was achieved by injecting 1 µM CR1(1-3) for 3 min and 1 M NaCl for 30 s. For each C3b surface this convertase formation assay was repeated twice. The data were exported and depicted in OriginPro^®^. The dissociation rates of the convertase were compared by fitting the dissociation curve using the exponential decay function (ExpDec1) of the OriginPro^®^ evaluation software. The response data for the interval between 200 and 780 s after the start of the FB and FD injection was used for the fit.

### Fluid-phase factor I susceptibility assay

The fluid-phase FI susceptibility assay is a variation of a standard cofactor assay. It was conducted as previously described with small modifications (28–30). Briefly, 2 µg of C3b, 10 nM or 100 nM of FI and 100 nM or 1 µM of CR1(15-17) were mixed with PBS and incubated at 37°C for up to 5 h on a rotary shaker at 1200 rpm. The reactions were stopped by adding reducing SDS-PAGE sample buffer, after which the samples were incubated at 95°C for 3 min. The samples were subsequently analyzed on 9% SDS-PAGE gels. C3b from Complement Technology and one sample of C3b from donor D1 that had been frozen and thawed for a total of five times were analyzed. The gels were stained with Coomassie Brilliant Blue R250 (Sigma-Aldrich) and the densitometric analysis of the bands was performed using the software ImageJ after imaging with an Amersham Imager 680. The grey value of the α’-band (at 115 kDa) was normalized to the grey value of the β-band (at 75 kDa) and depicted in OriginPro^®^ evaluation software.

### Western blot for the detection of iC3b

1 µg of C3b from different batches and 25-1,000 ng of iC3b (Complement Technology) were applied to a 9% SDS-PAGE gel. The proteins were then transferred onto an Amersham™ Hybond™ low fluorescence 0.2 µm PVDF membrane (Cytiva) at 30 V for 1 h. The membrane had been activated in methanol for 1 min and washed with distilled water and Towbin buffer. The membrane was then washed with PBS and blocked with 5% milk powder in PBS at ambient temperature for 2 h. Polyclonal goat anti-human C3 serum (Complement Technology) was used as the primary antibody source at a dilution of 1:5,000 in 5% milk powder in PBS. The membrane was washed twice with PBST (PBS supplemented with 0.05% Tween20) and once with PBS. Then, it was stained with mouse anti-goat IgG-CFL 647 (sc-516244, Santa Cruz Biotechnology) at a dilution of 1:2,000 in 5% milk powder in PBS, after which it was washed with PBST and PBS. Densitometric analysis of the bands was performed using ImageJ software after imaging with an Amersham Imager 680. To detect iC3b, the signal of the band at approx. 50 kDa (C3α’-46 kDa) was measured and depicted using OriginPro^®^ evaluation software.

### Anti-human FI polyclonal antibody activity in cofactor assay

2 µg of C3b (an equimolar mixture of D1-D3) were incubated with 40 nM FI (equivalent to a 10% serum concentration), 400 nM CR1(15-17) and anti-human FI pAb (15.6-2,000 nM) for 4 h at 37°C. The reaction was stopped by the addition of a reducing buffer containing 2-mercaptoethanol, after which the samples were incubated at 95°C for 3 min. The samples were loaded onto a 9% SDS-PAGE gel. As positive control, PBS was added instead of the anti-human FI pAb and as negative control, no FI was added. Densitometric analysis of the C3α’-46 kDa band was performed using ImageJ. To define the peak bases against the background noise, straight lines were drawn across the base of the peaks and the area of the peaks was measured. The diagram of the determined peak areas of the C3α’-46 kDa band was displayed using the OriginPro^®^ evaluation software. The area of the marker lane that was free of any bands at the relevant height was used as the background signal.

## Results

**Figure 1:**
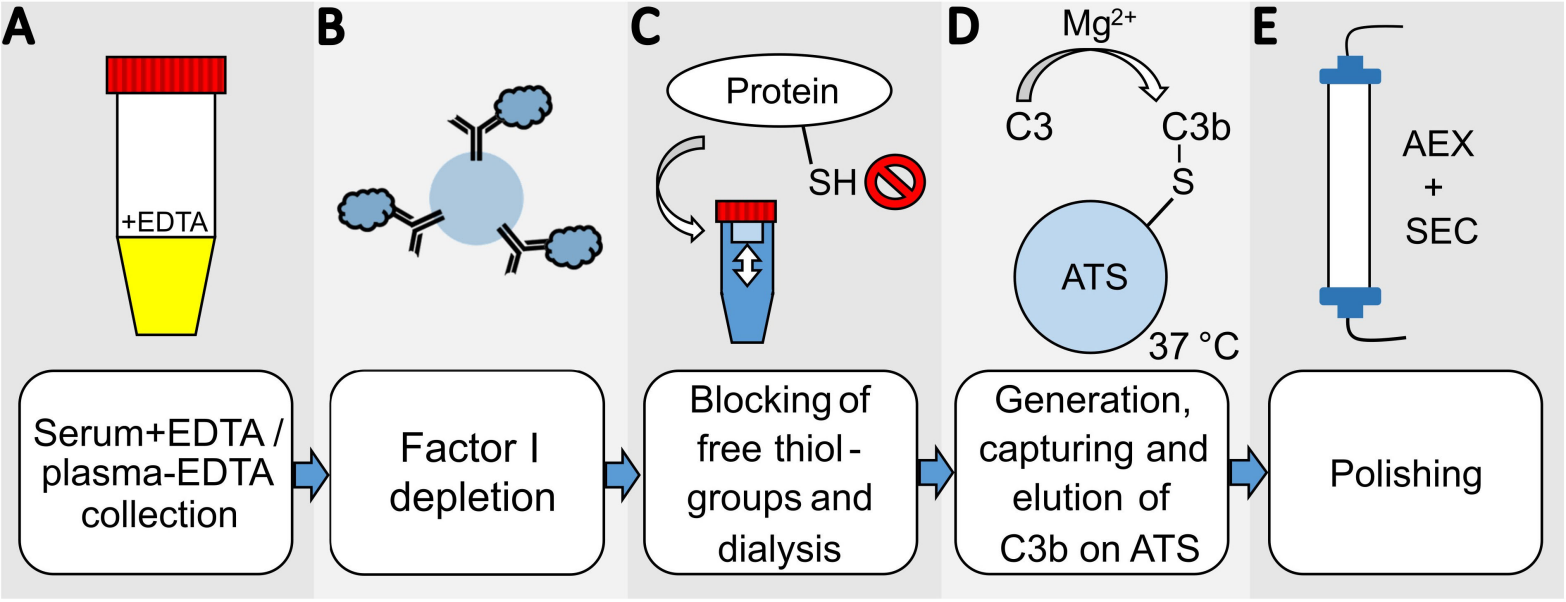
Overview scheme of the novel C3b-purification-method. **(A)** Serum with EDTA or plasma-EDTA from one donor is collected and **(B)** incubated with anti-FI polyclonal antibodies coupled to CNBr-activated Sepharose beads for FI-depletion. **(C)** The cysteines of all proteins in the FI-depleted serum/plasma are blocked by iodoacetamide, and the buffer is then exchanged by dialysis for PBS supplemented with 5 mM EDTA. **(D)** 10 mM MgEGTA and activated thiol-sepharose beads (ATS) are added to activate the alternative pathway of the serum/plasma and directly capture C3b. The C3b is eluted using 1 mM cysteine in PBS in several elution-cycles. The eluted protein is diluted with 20 mM phosphate buffer at pH 7.4 for **(E)** further purification by anion-exchange chromatography (AEX). The final polishing step and buffer exchange is performed by size-exclusion chromatography (SEC).

**Figure 2:**
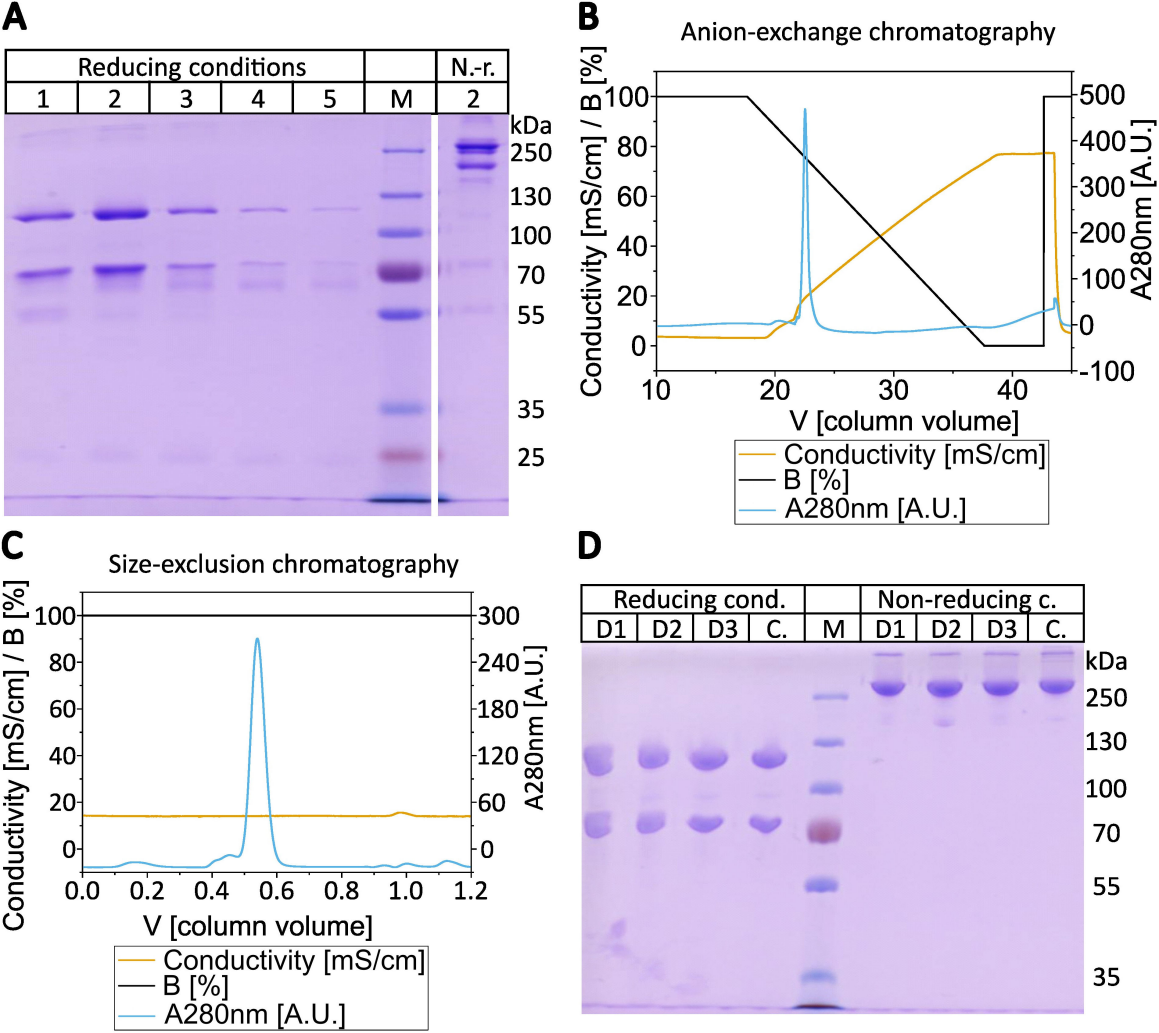
Purification of C3b. **(A)** SDS-PAGE of the consecutive elution steps from activated thiol-Sepharose (ATS) beads. In total five elution steps with 1 mM cysteine in PBS were performed, which were analyzed under reducing (2-mercaptoethanol) conditions and elution step 2 was also applied under non-reducing (*i.e.* absence of 2-mercaptoethanol) conditions. The elution of C3b from ATS-beads of donor D1 is shown as a representative gel of the elution fractions. **(B)** Chromatogram from the subsequent anion-exchange chromatography using MonoQ 10/100 GL. C3b elution started at approx. 22% elution buffer (16 mS/cm at 4°C, approx. 220 mM NaCl) at pH 7.4. The anion exchange chromatogram of the ATS-eluted C3b of donor D3 is shown as a representative chromatogram. **(C)** Chromatogram of the size-exclusion chromatography with Superdex 16/600–200 pg in PBS. Elution started at approx. 0.5 CV. The size-exclusion chromatogram of C3b eluted from anion-exchange step of donor D1 is shown as a representative chromatogram. **(D)** SDS-PAGE of purified C3b from three different donors (D1-D3) compared to commercial C3b **(C)**. 2 µg of C3b were loaded onto each lane in SDS-loading buffer under reducing or non-reducing conditions (presence or absence of 2-mercaptoethanol). Molecular weight marker as indicated.

In order to develop an efficient and direct method for purifying C3b from plasma or serum preparations, we adapted a published protocol describing the purification of iC3b from plasma/serum using activated thiol-Sepharose (ATS) (31–33). An additional step was incorporated into the procedure to deplete plasma/serum from the protease FI (**Figure 1**). This is achieved by incubating the plasma/serum with affinity-purified anti-FI antibodies immobilized on Sepharose beads, in the presence of EDTA which blocks any complement activity. In instances where EDTA plasma is utilized as the C3 source, the addition of EDTA is not required. Before immobilizing the affinity-purified polyclonal anti-FI antibody onto Sepharose beads, we tested its binding and neutralizing activity towards FI and found it to be ample (**Supplementary Figure S1**). After FI depletion using the generated anti-FI resin, all free thiol groups in plasma/serum are blocked with iodoacetamide. Subsequently, the excess of iodoacetamide is removed by dialysis. Then magnesium ions are introduced into the system to exceed the EDTA concentration. This enables the alternative pathway (AP) to be initiated and the obtained plasma is exposed to ATS beads at 37°C to activate the AP. Upon conversion of C3 by AP convertase into C3b, the thioester domain swings outward, exposing the thioester moiety, which subsequently undergoes hydrolysis. This process results in the generation of a novel thiol group on C3b (Cys1010), which can be specifically captured by the thiol-reactive ATS-Sepharose via the formation of a disulfide bond between the sulfhydryl group of the thioester moiety of C3b and ATS (34). Comparing the existing protocol for capture and elution of iC3b from ATS beads (33), we reduced the concentration of the eluting agent L-cysteine to 1 mM, as higher cysteine concentrations hold the risk of interfering with disulphide bridge integrity. SDS-PAGE was performed to analyze the quality of the C3b eluted from ATS beads using 1 mM cysteine dissolved in PBS (**Figure 2A**). The C3b preparations were analyzed under reducing and non-reducing conditions and revealed the expected bands for the C3b α- and β-chain (at approximately 110 and 70 kDa, respectively), as well as a major band at the height of the 250 kDa size marker. The most prominent bands correspond to the expected bands of the C3b protein, with only a few faint contaminating bands visible, suggesting that the ATS capture of C3b directly from FI-depleted plasma represents a very efficient first purification step. All C3b-containing fractions were pooled and subjected to anion-exchange chromatography. As expected, C3b eluted in one major peak from the anion-exchange resin (**Figure 2B**). Using size-exclusion chromatography, the buffer was exchanged for PBS and the C3b-preparation further purified (**Figure 2C**). The chromatogram demonstrates that this polishing step led to the elimination of minor quantities of contaminating proteins with hydrodynamic radii that differ from those of C3b. This resulted in pure C3b preparations with an overall purity of around 90%, as determined by densitometry (**Figure 2D**, **Supplementary Figure S2**). Incomplete removal of FI during the purification process could result in the formation of unwanted iC3b. Therefore, we checked for potential iC3b impurities using SDS-PAGE and western blot analysis to try to detect the C3α’-46 kDa band. No evidence of iC3b contaminations was found in our C3b preparations (**Supplementary Figures S3, S4**). With regard to yield, the direct and novel purification method used 14.0 to 14.5 ml of EDTA plasma for each donor D1, D2, and D3. This process yielded 2.9, 2.3, and 2.1 mg of pure and functional active C3b, respectively.

**Figure 3:**
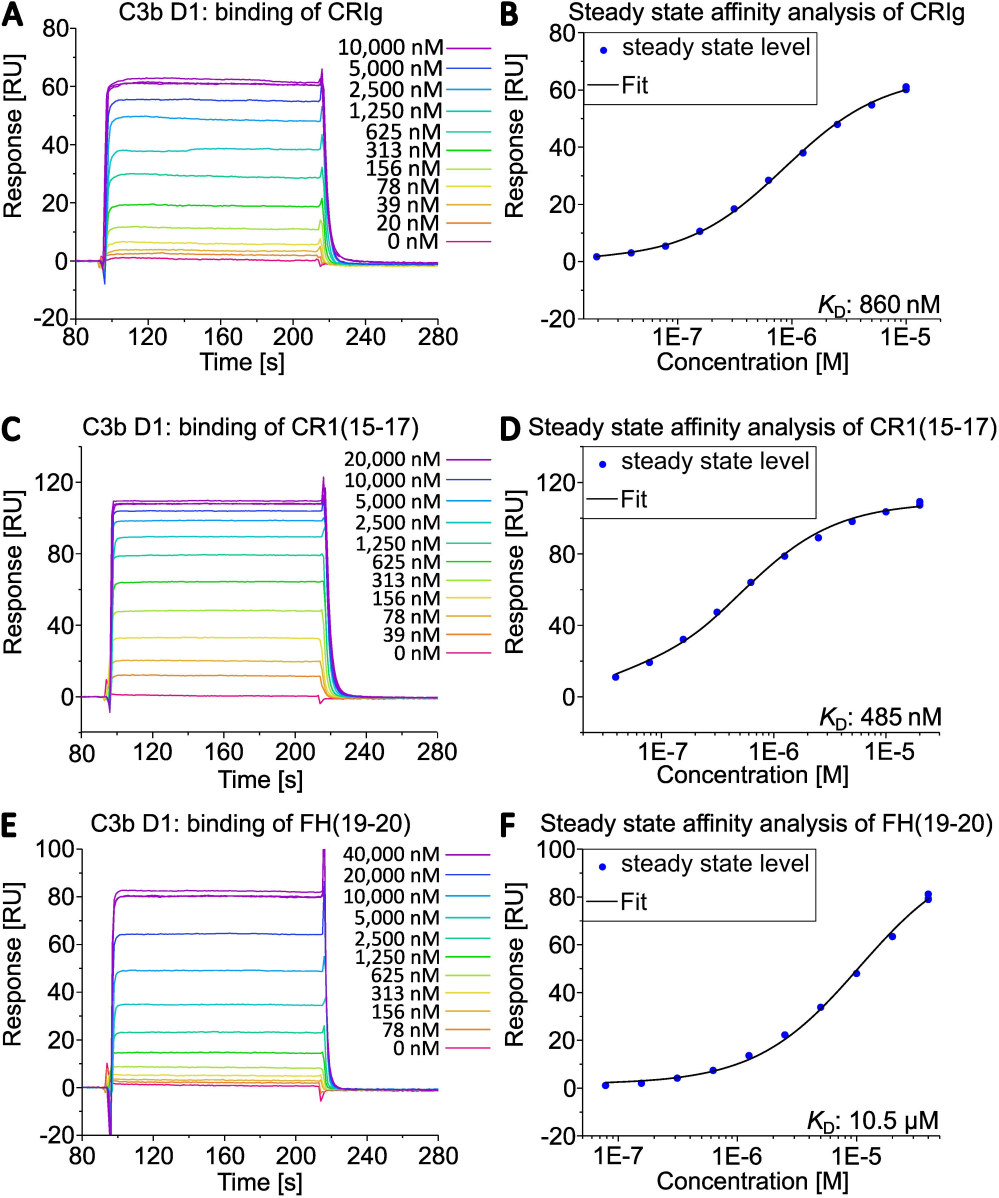
Interaction of C3b from D1 with different complement regulators assessed by SPR. C3b of donor D1 was specifically biotinylated at the sulfhydryl of the thioester moiety and immobilized on a streptavidin (SA) sensorchip (Cytiva). The binding of **(A)** CRIg, **(C)** CR1(15-17), **(E)** FH(19-20) was measured. Steady state affinity fits returned the equilibrium dissociation constants by applying a 1:1 binding model **(B, D, F)** in the Biacore X100 evaluation software.

**Figure 4:**
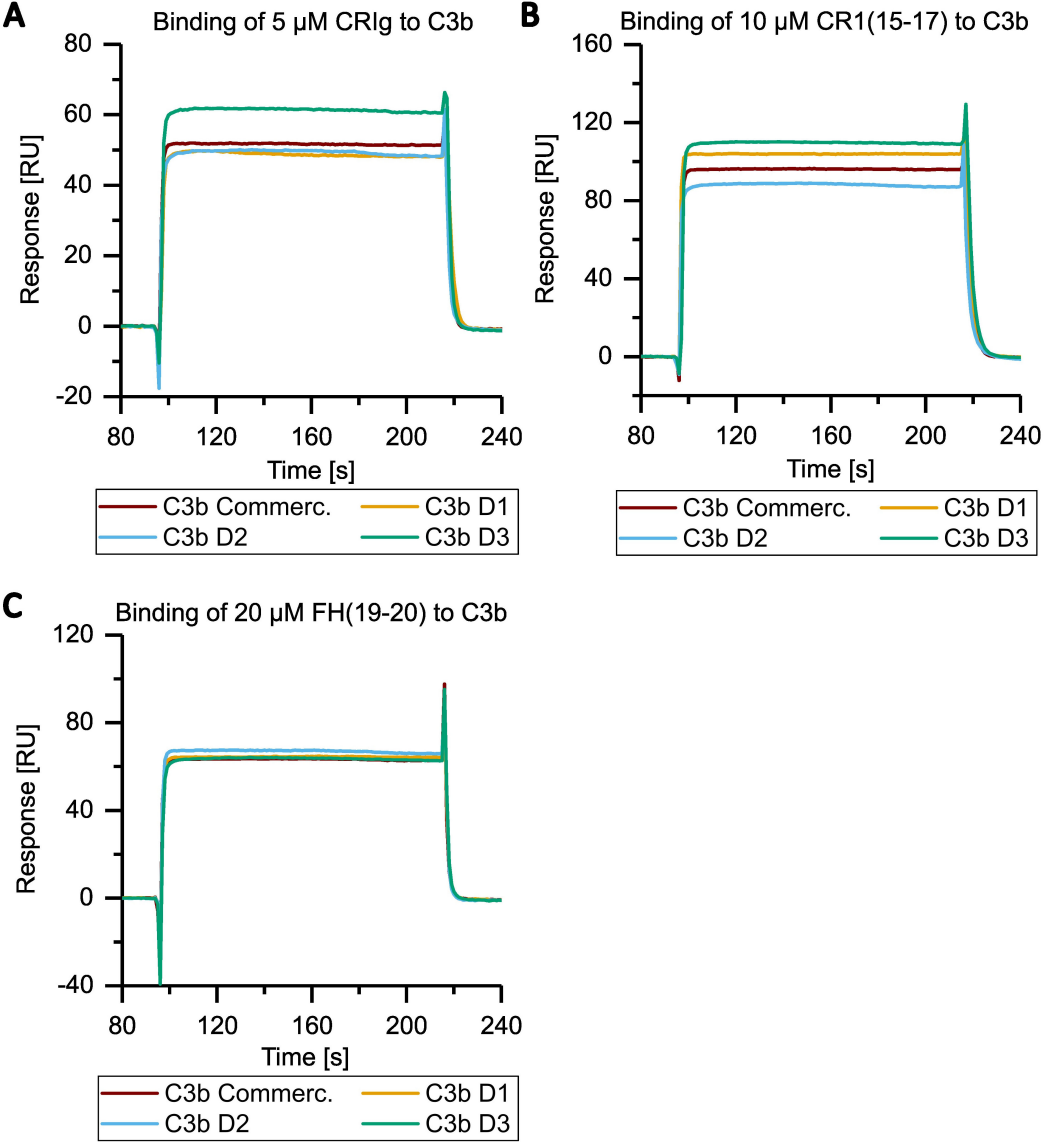
Overlay of SPR analysis of the interaction of C3b from different donors to CRIg, CR1(15-17), FH(19-20) at a specific concentration. Panels **(A–C)** contain selected data from **Figure 3** and **Supplementary Figures 5–7**. Purified and biotinylated C3b from the three individual donor batches and commercial C3b were immobilized as described in **Figure 3**. **(A)** The binding of C3b from all donors and from commercial source to 5 µM of CRIg is shown. The same is depicted for the binding to **(B)** 10 µM CR1(15-17), **(C)** 20 µM FH(19-20).

Next, we compared the activities of commercially available C3b (a mixture of C3b molecules from multiple donors) and individual C3b preparations from three healthy donors that had been purified using the novel method. First, we analyzed the binding behavior of several known ligands that bind to C3b using SPR. C3b was specifically biotinylated at the thioester moiety to enable immobilization in physiological orientation on a streptavidin-coated sensorchip. Concentration series of three different recombinantly expressed C3b ligands were then applied to each immobilized C3b surface. The applied ligands were CRIg, FH(19-20), and CR1(15-17). They bind to different non-overlapping surface patches on C3b spanning the macroglobulin (MG) domains 3-6, thioester domain (TED), and complement C1r/C1s, Uegf, Bmp1 (CUB) domain -TED-MG2-MG4-MG6-MG7, respectively (35–37). Thus, this approach allows for a broad functional evaluation of the purified C3b preparations (**Figures 3** and **4**, **Supplementary Figures S5–S7**). The binding affinities of the three tested ligands to commercially available pooled C3b and C3b from three individual donors are very similar (**Table 2**). Nonetheless, slight differences are measurable, which is to be expected given the genetic variability among donors, very small differences in protein quality, and variations in C3b immobilization levels on the SPR sensorchips (which were less than 10%). As demonstrated in **Figure 4**, the binding of CRIg and CR1(15-17) to distinct C3b preparations manifests greater variability than the binding of FH(19-20), for which all four responses exhibit a high degree of similarity.

**Table 2:** Summary of functional analyses of commercial C3b and C3b purified using the direct, novel protocol.

Determined parameters	C3b (commercial)	C3b D1	C3b D2	C3b D3
Affinity for CRIg	754 nM	860 nM	944 nM	795 nM
Affinity for CR1(15-17)	476 nM	485 nM	650 nM	438 nM
Affinity for FH(19-20)	9.3 µM	10.5 µM	10.4 µM	10.6 µM
Estimated natural decay of the convertase C3bBb	1.9 * 10^-3^ s^-1^	2.0 * 10^-3^ s^-1^	2.0 * 10^-3^ s^-1^	1.9 * 10^-3^ s^-1^
Fluid-phase cofactor activity: percentage of α’-chain band remaining (in respect to β-chain band)	32.7 %	8.7 % (30.2 % after a total of five freeze-thaw cycles)	8.4 %	7.1 %

**Figure 5:**
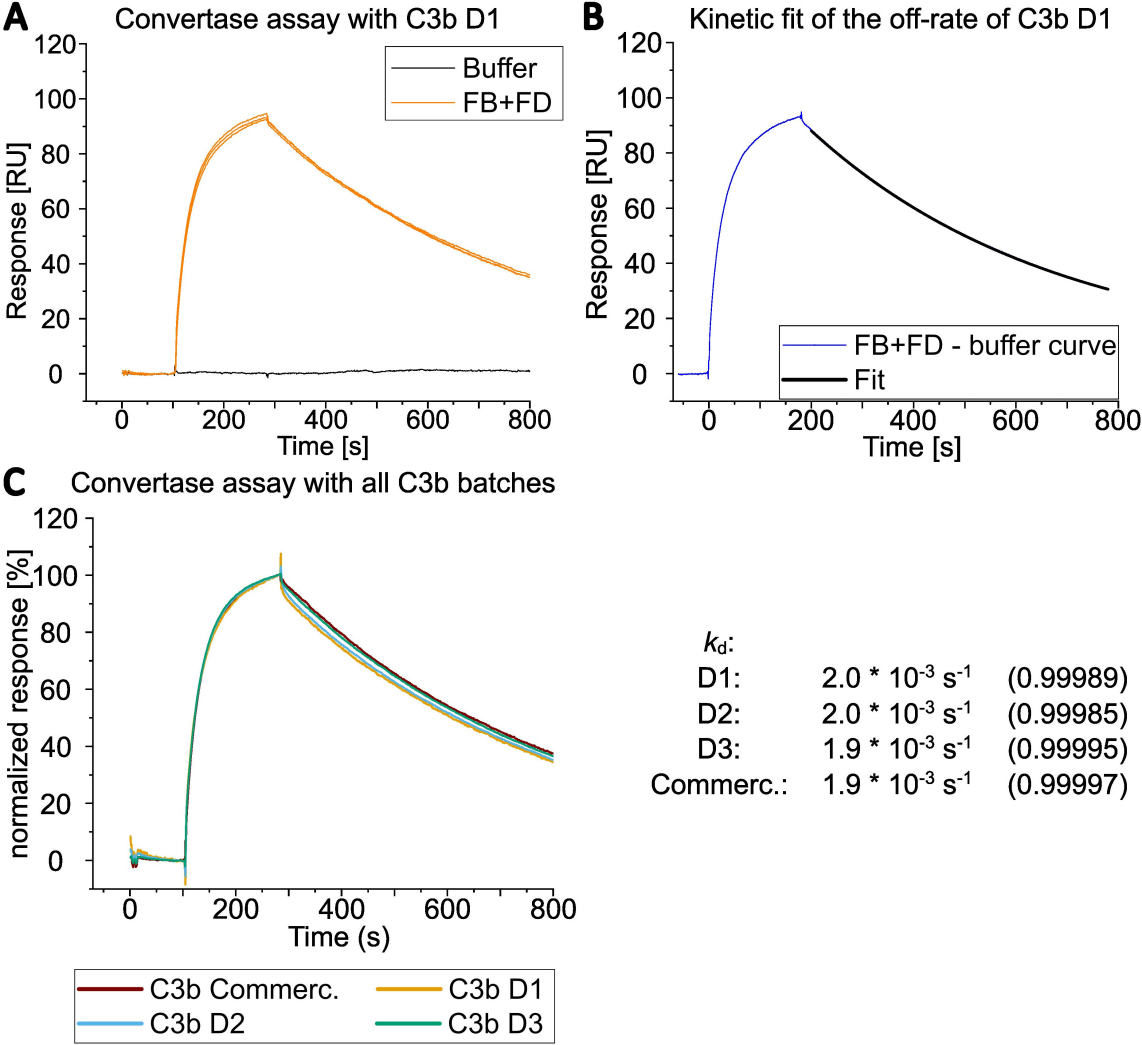
Overlay of SPR analysis of convertase assembly and dissociation. **(A)** Convertase formation by SPR was measured by simultaneously injecting 600 nM FB and 100 nM FD onto C3b from D1 immobilized via the thioester moiety (biotinylated sulfhydryl). Regeneration of the surface was achieved by applying CR1(1-3) at 1 µM followed by an injection of 1 M NaCl (regeneration steps are not shown in the graph; see **Supplementary Figure S8** for sensorgrams including regeneration with CR1(1-3)). The FB and FD injections were performed in total three times and are shown as overlay of three curves. **(B)** Only one of three curves from *A)* and its corresponded curve fit is shown after the buffer curve had been subtracted (injection start of FB and FD was set to 0 seconds). The natural convertase dissocation rate (*k*_d_) was estimated by applying an exponential decay fit with OriginPro^®^ evaluation software between 200 to 780 s after injection of FB and FD was started. **(C)** Convertase formation on all four C3b preparations with 600 nM FB and 100 nM FD. Only one of three curves is shown for each donor C3b. Responses were normalized to allow for better comparison. The dissociation rates of bimolecular convertase C3bBb were estimated by fitting the dissociation part of the sensorgram with the OriginPro^®^ evaluation software as in *B)*. The dissociation rates were calculated for all three curves per donor and their values (per donor) were averaged. The average of the R^2^ of the fits per donor is displayed in parenthesis.

Subsequently, the sensorchip-immobilized C3b surfaces were further utilized to characterize the functionality of the different C3b preparations with regard to convertase formation and decay (**Figure 5**, **Supplementary Figure S8**). The on-chip convertase assay showed comparable behavior in C3bBb formation and decay for all four different C3b preparations.

**Figure 6:**
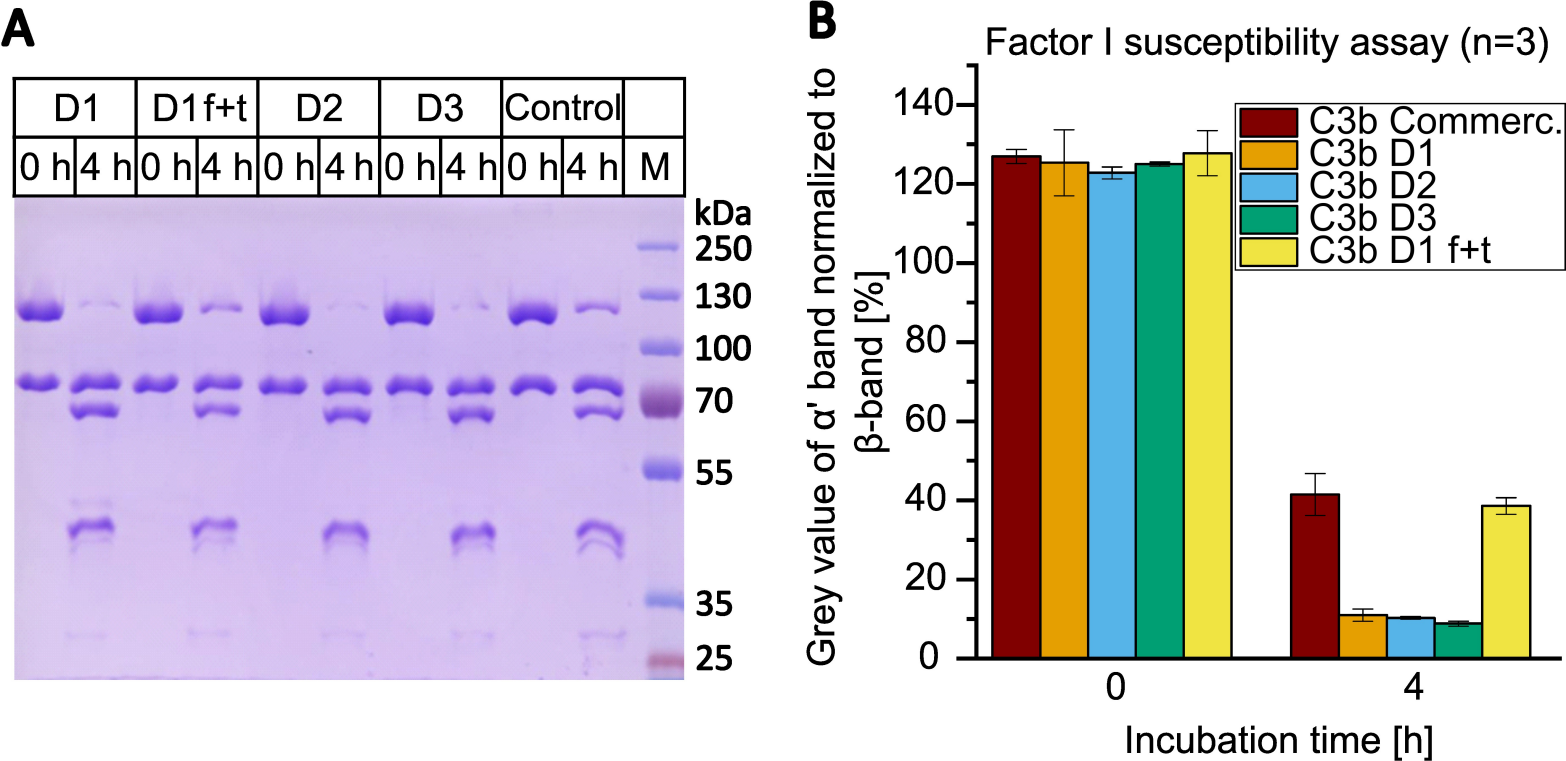
Factor I susceptibility assay with C3b from different donors (D1-3) compared to commercial C3b (control). **(A)** C3b was incubated with 10 nM FI and 100 nM CR1(15-17) at 37°C for 0 or 4 h. Samples were reduced, heat-inactivated and applied to 9% SDS-PAGE gel. C3b preparations D1, D2 and D3 were once frozen and thawed prior being assayed. In addition, C3b from D1 was repeatedly frozen (in liquid nitrogen) and thawed for a total of five cycles and then tested in the assay (D1 f+t). Line M contains the molecular weight marker. Corresponding sizes are indicated. **(B)** Average results of three assays. The grey value of the α’-band (at 115 kDa) was normalized to the grey value of the β-band (75 kDa) using ImageJ and Microsoft Excel. The values of three independent assays were averaged. Results are shown as bars with standard deviation being indicated.

The susceptibility of the C3b batches to proteolytic regulation by FI was evaluated using a fluid-phase FI susceptibility assay (**Figure 6**). In the case of the home-made C3b preparations, the bands corresponding to the α’-chain were found to be almost entirely proteolytically processed into the smaller α’-chain fragments. In contrast, the processing of the α’-chain of the commercially available C3b by FI was found to be substantially less efficient. To test whether the reduced susceptibility to FI of commercial C3b could be ascribed to alterations induced, for example, by freeze-thaw cycles, we submitted C3b from donor D1 to five freeze-thaw cycles and subsequently reanalyzed the susceptibility to proteolytic regulation by FI. Freeze-thaw cycles did indeed induce some resistance to regulation by FI in a substantial proportion of C3b in this batch of donor D1 (**Figure 6**). We then tested whether freshly purified C3b, which had not been submitted to a single freeze-thaw cycle, would be fully receptive to proteolytic regulation by FI, which would result in complete processing of the α’-chain band of C3b. Therefore, C3b from a fourth donor was purified according to the new protocol and immediately subjected to our fluid phase FI susceptibility assay. We intentionally overloaded the gel to sensitively assess the processing of the C3b α’-band (**Supplementary Figure S9**). The α’-band of the fresh C3b preparation was found to be fully processed by FI, indicating that a single freeze-thaw cycle can affect a proportion of molecules in a C3b preparation. Finally, we tested if the FI that was obtained from the FI depletion step retains functional activity. The functionality of FI eluted from the home-made anti-FI affinity column was confirmed by a fluid-phase FI functional assay (**Supplementary Figure S10**).

## Discussion

A novel purification protocol has been developed for the direct isolation of C3b from plasma or serum sources, eliminating the necessity for any intermediate purification of C3. Our protocol does not offer any advantages when it comes to studying the unique functions of native C3, since it produces purified C3b directly. However, the novel method is rapid (three days) and yields substantial amounts of high-quality C3b in only six purification steps. From a source of approximately 14 ml of plasma/serum, 2 to 3 mg of fully functional C3b can be expected (approximately 15% yield considering a C3 plasma/serum concentration of 1.2 mg/ml). The new method has been shown to offer several advantages, including a rapid turnaround time, the elimination of the requirement for convertase reagents or enzymes to cleave C3 to C3b, and the capacity to purify C3b from low-volume blood sources. This latter capability is particularly advantageous for the study of C3b from patient samples. This is particularly helpful for analyzing the functional effects of mutations or single-nucleotide polymorphisms in C3b when only milliliter quantities of blood samples are available. When we compare our final yield of 15% with the yields of C3/C3b purifications reported in the literature, which range from 20% to 61%, it is clear that our yield of 15% is rather low. However, we obtained this yield from a starting volume of only 14 ml of plasma, whereas the values cited in the literature are from purification batches using 0.1 to 3 liters (18–20, 38, 39). The only protocol that describes C3/C3b purification from a starting volume below 5 ml does not specify a yield (22). Another advantage is C3b integrity and activity as all steps are performed under physiological pH, using PBS-like conditions throughout. This avoids high or low pH values, or the precipitants frequently employed in other C3/C3b purification protocols. However, a disadvantage of the novel method is that the blood source requires FI depletion in the first step. This requires the use of affinity-purified polyclonal antibodies against human FI, which are coupled to chromatography beads to remove FI by conservative gravity flow chromatography. However, as a concomitant effect, FI can be recovered, and the beads containing the polyclonal anti-FI antibody can be reused multiple times over the years. The removal of FI is essential for the effective operation of this protocol. Trace amounts of FI would be sufficient to convert a portion of the ATS-captured C3b into iC3b, which would reduce the yield and require separation of C3b and iC3b in sequential steps. A potential alternative to the prior removal of FI could be the addition of protease inhibitor that blocks the catalytic triad of FI. However, the proteolytic activity of FI is not blocked by typical serine protease inhibitors (40) or require pre-incubation of isolated FI and the substrate C3b prior addition of a cofactor (41). Such procedures are not feasible for a direct capture and purification step directly from blood sources. Both purification protocols, that of Scott and Fothergill (20) and ours, remove FI to stop C3b conversion into iC3b. However, FI removal according to the Scott and Fothergill protocol follows a completely different method. First, the typical sequential precipitation steps from serum or plasma with PEG are performed, followed by C3 conversion to C3b, and subsequent separation of C3b from remaining traces of FI by anion exchange chromatography. Thus, Scott and Fothergill undertake a preliminary purification of C3 from blood sources prior to its conversion to C3b through the utilization of the co-precipitated convertase components FB and FD. The advantage of this and our method is that no purified proteins need to be added for the processing of C3 to C3b. The protocol developed by Scott and Fothergill does, however, have certain disadvantages. These include the need for purified FH to prepare a C3b affinity column to capture the processed C3b, as well as non-physiological conditions during purification (such as precipitation and non-physiological buffer conditions). Nevertheless, this method is similarly fast to ours (three days of purification time) and requires only one additional step (seven instead of six). It was observed that complete processing of the C3b α’-chain by FI is only achieved with freshly purified C3b that has not been subjected to freeze-thaw cycles. Even long incubation times or excessive reagents could not overcome the resistance of the C3b α’-chain to proteolytic processing following the freezing of the C3b preparation. The observation that a proportion of the C3b molecules are resistant to degradation by FI is consistent with numerous literature reports employing a comparable assay setup (30, 42–49). Freeze-thaw cycles speculatively induce structural changes in a portion of the C3b molecules, thus causing this resistance to FI regulation. In summary, we describe the first direct method for obtaining pure and active C3b directly from blood sources such as plasma/serum in only six steps, thus paving the way for rapid and economical purification of C3b from plasma/serum sources, even when only limited sample volumes are available.

## Data Availability

The original contributions presented in the study are included in the article/**Supplementary Material**. Further inquiries can be directed to the corresponding author.
