# Supplementary material for: A novel and rapid method to purify the human complement opsonin C3b from human plasma
Source: Front Immunol. 2025 Aug 26;16:1639735. doi: 10.3389/fimmu.2025.1639735 (PMC12417186; doi:10.3389/fimmu.2025.1639735)
Supplement: Supplementary file 1 [file DataSheet1.pdf]

SUPPLEMENTAL MATERIAL online

A novel and rapid method to purify the human complement opsonin C3b from human plasma

Jannik Sichau[1] and Christoph Q. Schmidt[1]

[1]Institute of Experimental and Clinical Pharmacology, Toxicology and Pharmacology of Natural Products, University of Ulm Medical Centre, Ulm, Germany.

*** Correspondence:**
Christoph Q. Schmidt,

christoph.schmidt@uni-ulm.de

Supplemental Figures **(page 2-11)**

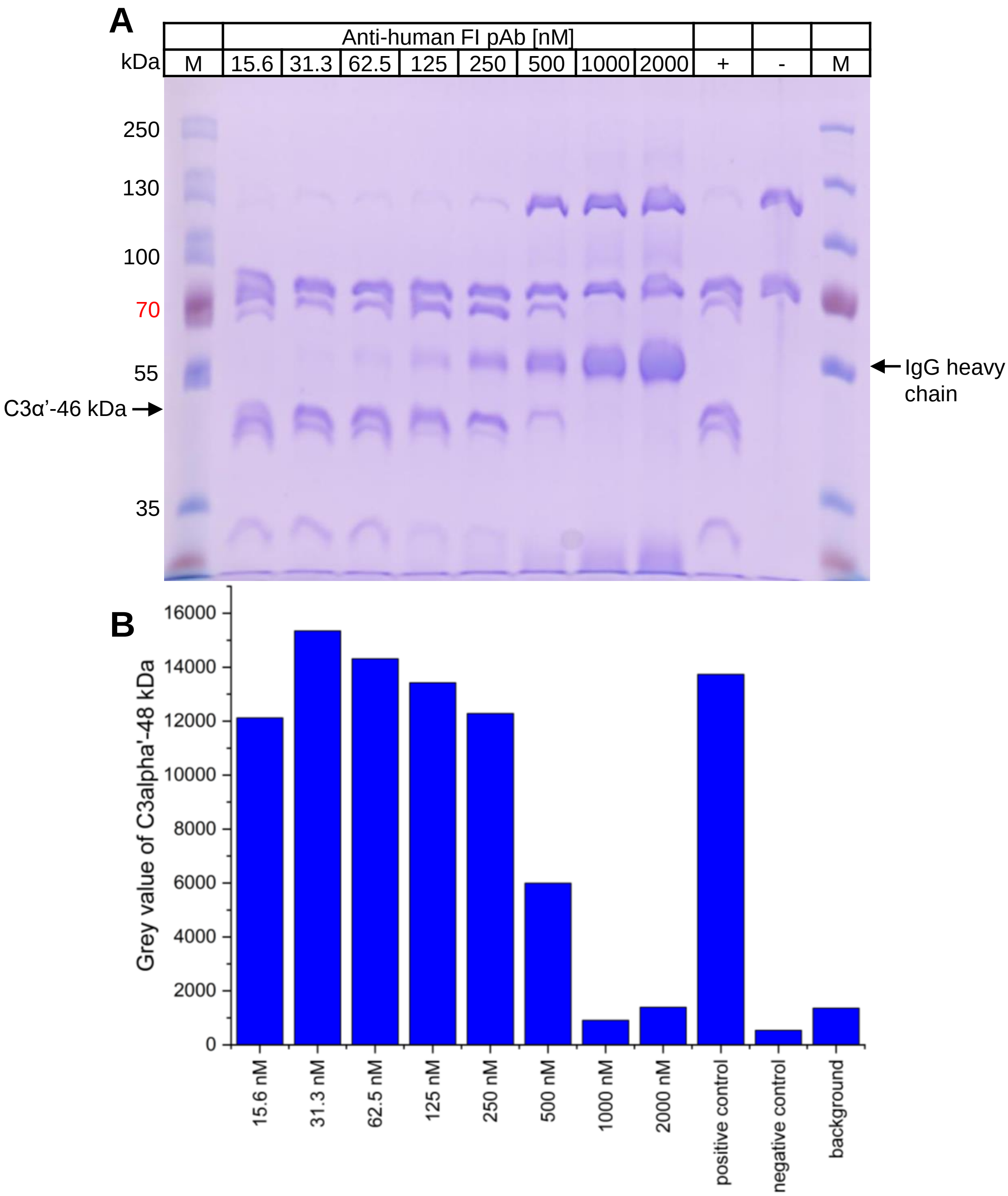

Supplemental Figure 1. Inhibition of Factor I activity by affinity-purified anti-human Factor I polyclonal antibody. A) 2 µg C3b (equimolar mixture of C3b from D1-D3) was incubated with 40 nM FI (corresponding to 10 % serum concentration), 400 nM CR1(15-17) and the indicated concentrations of affinity-purified anti-human FI pAb for 4 h at 37 °C. The reaction was stopped by adding 2-mercaptoethanol followed by incubation at 95 °C for 3 min. The samples were loaded onto a 9 % SDS-PAGE gel. PBS instead of pAb was included as a positive control. A sample without FI served as negative control. Molecular weight markers as indicated. **B)** Densitometric analysis of the C3α'-46 kDa band (height indicated by an arrow in *A)*) with ImageJ. To define the peak bases against the background noise, straight lines were drawn across the base of the peaks and the area of the peaks was measured (see *Supplemental Figures 2-4* for reference). The graph of the determined band areas of the C3α'-46 kDa band was displayed in OriginPro® evaluation software. The corresponding area of the marker lane, which is void of any band, was used as background signal.

A

B

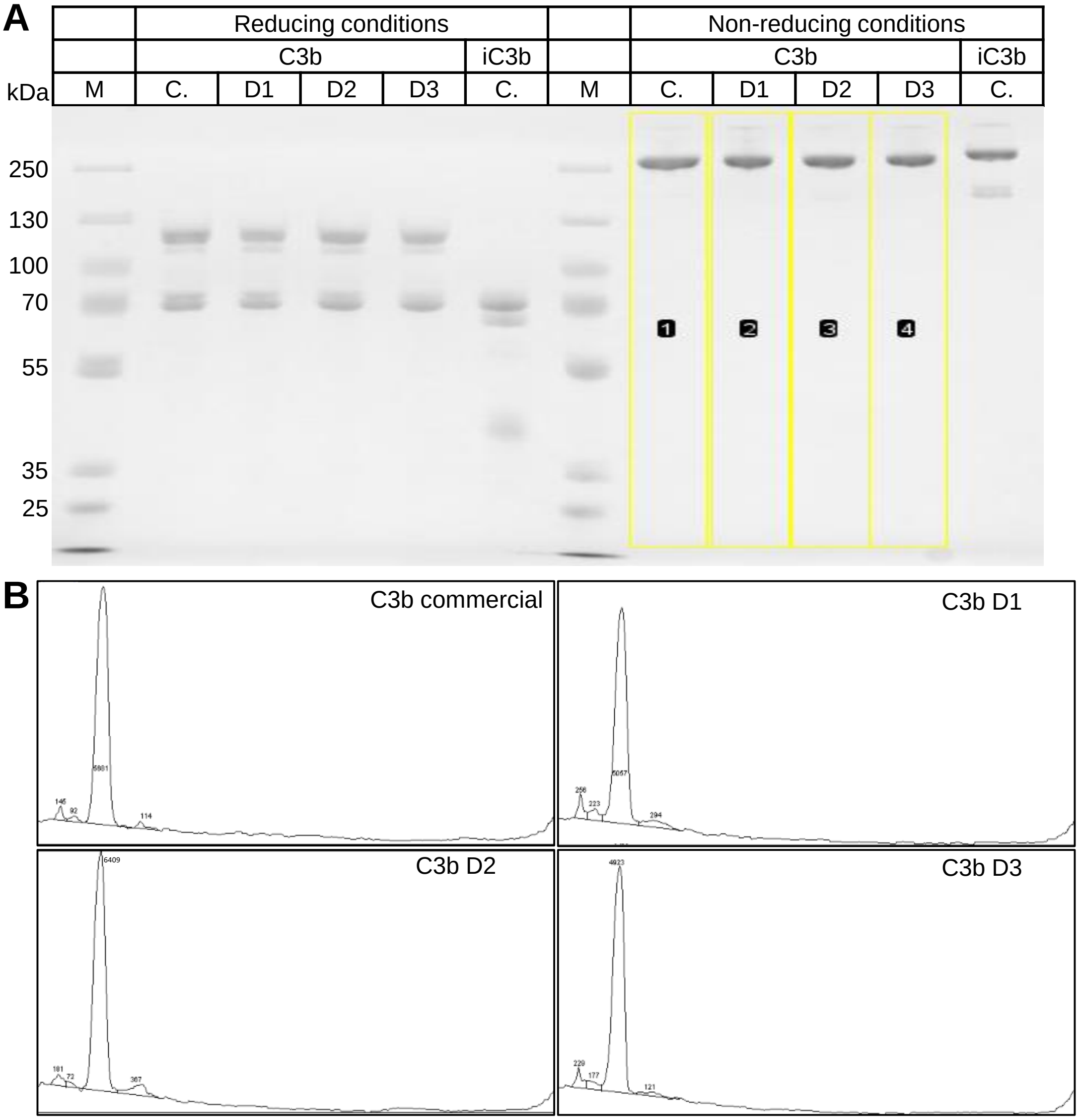

C

Densitometric peak area [%]	D1	D2	D3	C.
C3b monomer	86.7	89.7	90.3	94.2
C3b dimer	4.4	3.0	4.2	2.4
Total C3b	91.1	92.7	94.5	96.6
Residual	8.9	7.3	5.5	3.4

Supplemental Figure 2. Determination of C3b purity. A) SDS-PAGE of C3b purified from different donors analysed under reducing and non-reducing conditions. 1 µg of C3b from D1-D3 and Complement Technology (C.) were loaded onto a 9 % SDS-PAGE gel. In addition, 1 µg of commercial iC3b (Complement Technology) was loaded. The areas marked by yellow boxes were used for the determination of C3b purity. Molecular weight markers as indicated. **B)** Densitometric analysis of the areas in the yellow boxes. To define the peak base against the background noise, a straight line was drawn across the base of the peaks. **C)** Summary of densitometric analysis. The areas of the peaks were quantified and listed for C3b monomer (3rd peak from the left), C3b dimer (1st peak from the left) and residual signals (2nd and 4th peaks from the left).

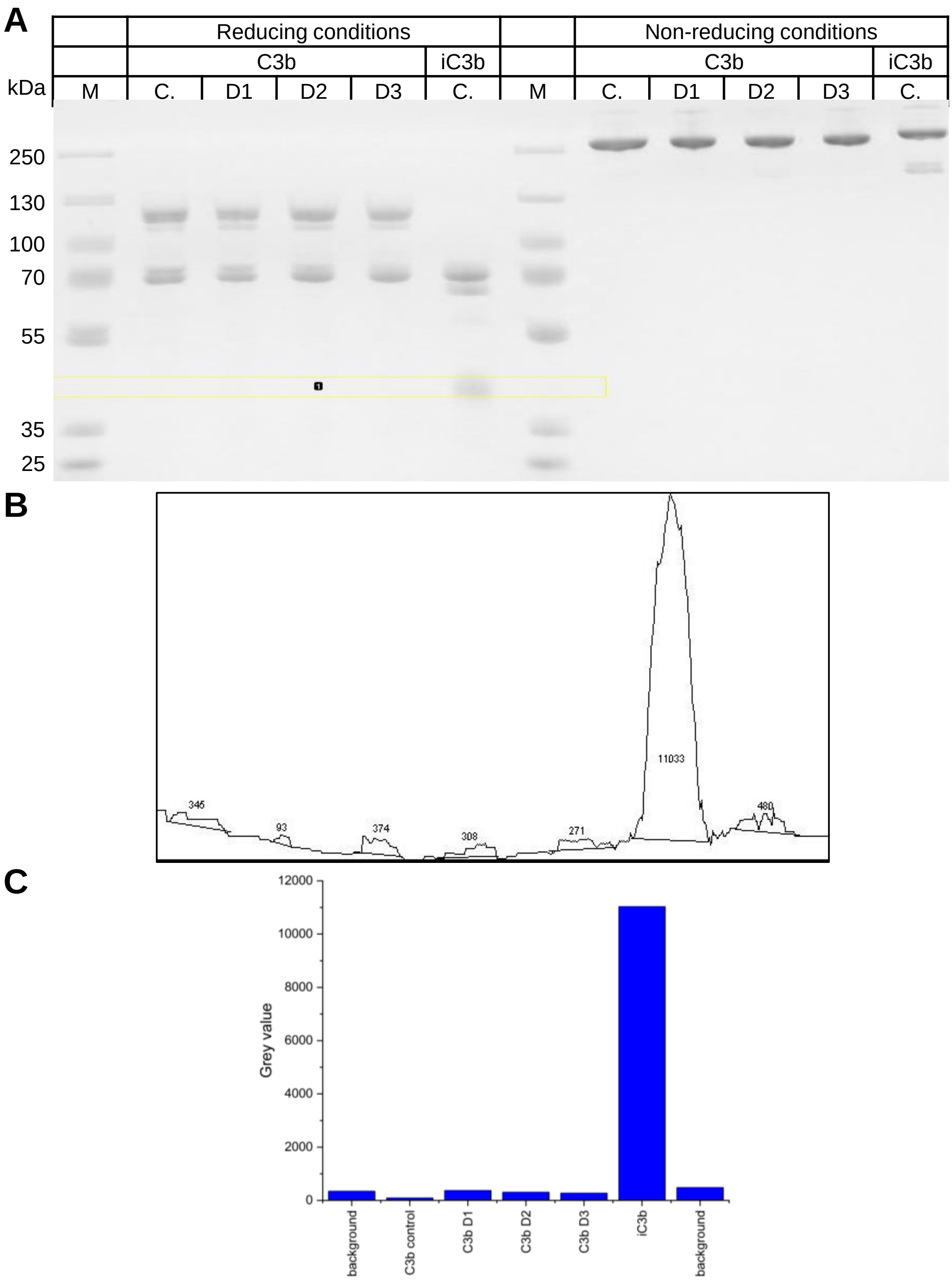

Supplemental Figure 3. Determination of potential iC3b contamination. A) The same gel from *Supplemental Figure 2* was used again, but this time for the densitometric analysis of iC3b. The areas marked by yellow boxes were used to determine potential iC3b impurities. Molecular weight markers as indicated. **B)** Densitometric analysis of the areas in the yellow boxes. To define the peak base against the background noise, a straight line was drawn across the base of the peaks. **C)** The area was measured and the area depicted as a bar graph.

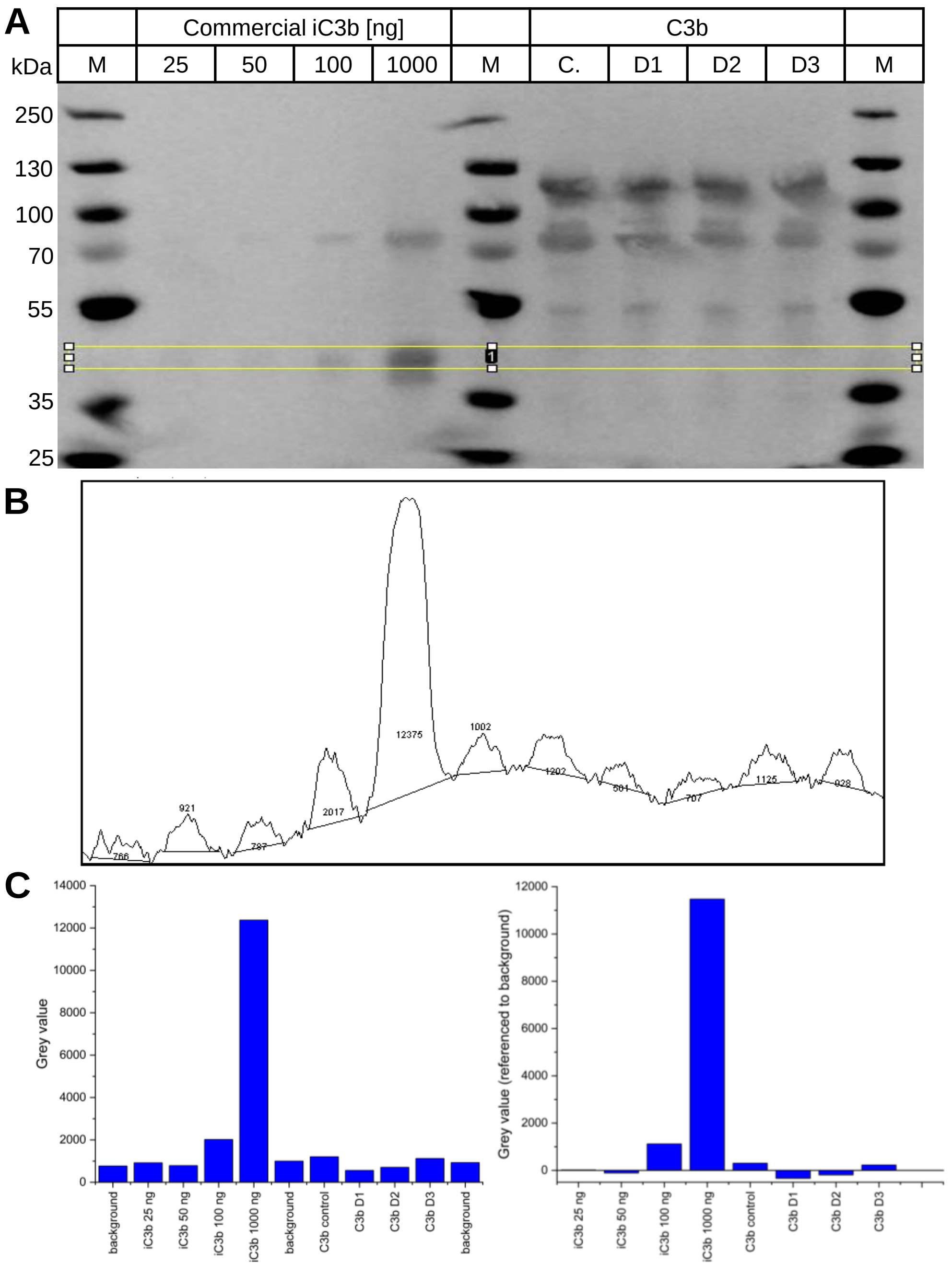

Supplemental Figure 4. Western blot for determination of potential iC3b contamination. A) 1 µg C3b from different donors (D1-D3) and from Complement Technology (C.) was loaded onto a 9 % SDS-PAGE gel. In addition, 25-1000 ng of commercial iC3b (Complement Technology) were added to the gel. The proteins were blotted onto a PVDF membrane and incubated with 1:5000 diluted goat anti-human C3 antiserum (Complement Technology), followed by 1:2000 diluted anti-goat IgG-CFL 647 (Santa Cruz Biotechnology). The level of the iC3b α'-46 kDa band used to quantify iC3b is indicated in the yellow box. Molecular weight marker as indicated. **B)** Densitometric analysis of the C3α'-46 kDa band with ImageJ. To define the peak bases against the background noise, straight lines were drawn across the base of the peaks and the area of the peaks were measured. **C)** Diagram of the determined band areas of the C3α'-46 kDa band. The marker with no band at the corresponding height was used as background signal and was subtracted (right graph).

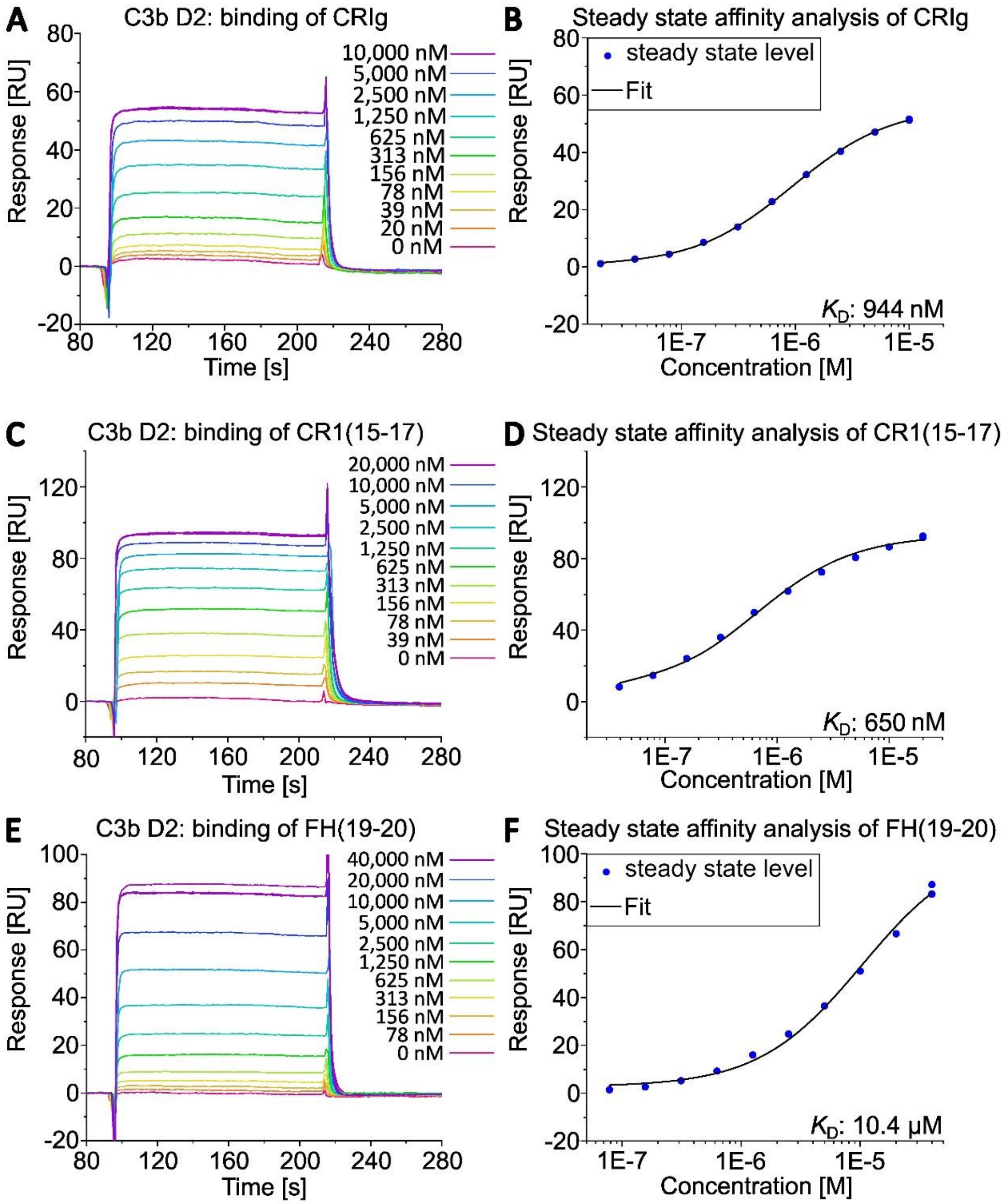

Supplemental Figure 5. Interaction of C3b from D2 with different complement regulators assessed by SPR. C3b of donor D2 was specifically biotinylated at the sulfhydryl of the thioester moiety and immobilised on a streptavidin (SA) sensorchip (Cytiva). The binding of **A)** CRIg, **C)** CR1(15-17), **E)** FH(19-20) was measured. Steady state affinity fits returned the equilibrium dissociation constants by applying a 1:1 binding model (**B, D, F**) in the Biacore X100 evaluation software.

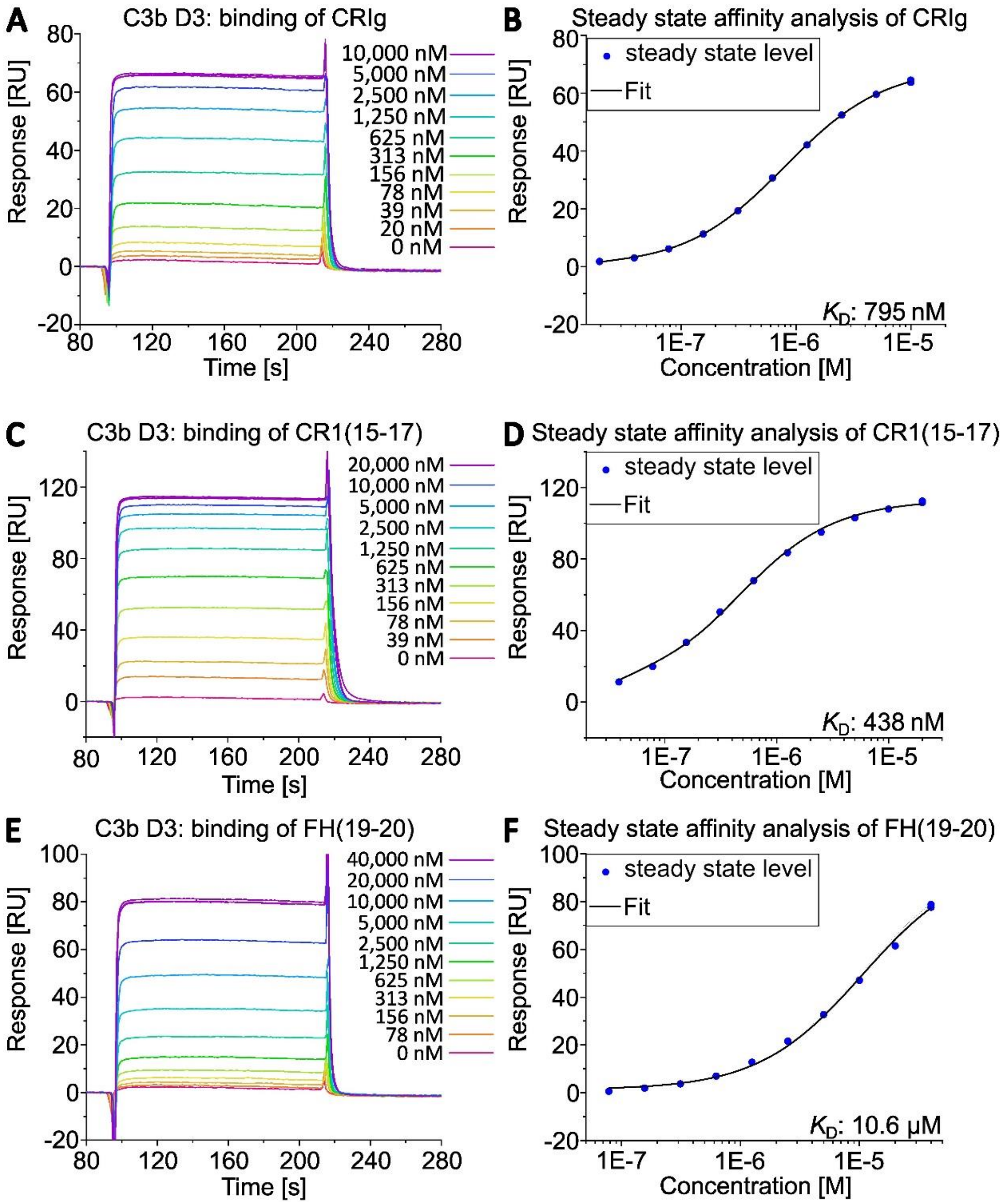

Supplemental Figure 6. Interaction of C3b from D3 with different complement regulators assessed by SPR. C3b of donor D3 was specifically biotinylated at the sulfhydryl of the thioester moiety and immobilised on a streptavidin (SA) sensorchip (Cytiva). The binding of **A)** CRIg, **C)** CR1(15-17), **E)** FH(19-20) was measured. Steady state affinity fits returned the equilibrium dissociation constants by applying a 1:1 binding model (**B, D, F**) in the Biacore X100 evaluation software.

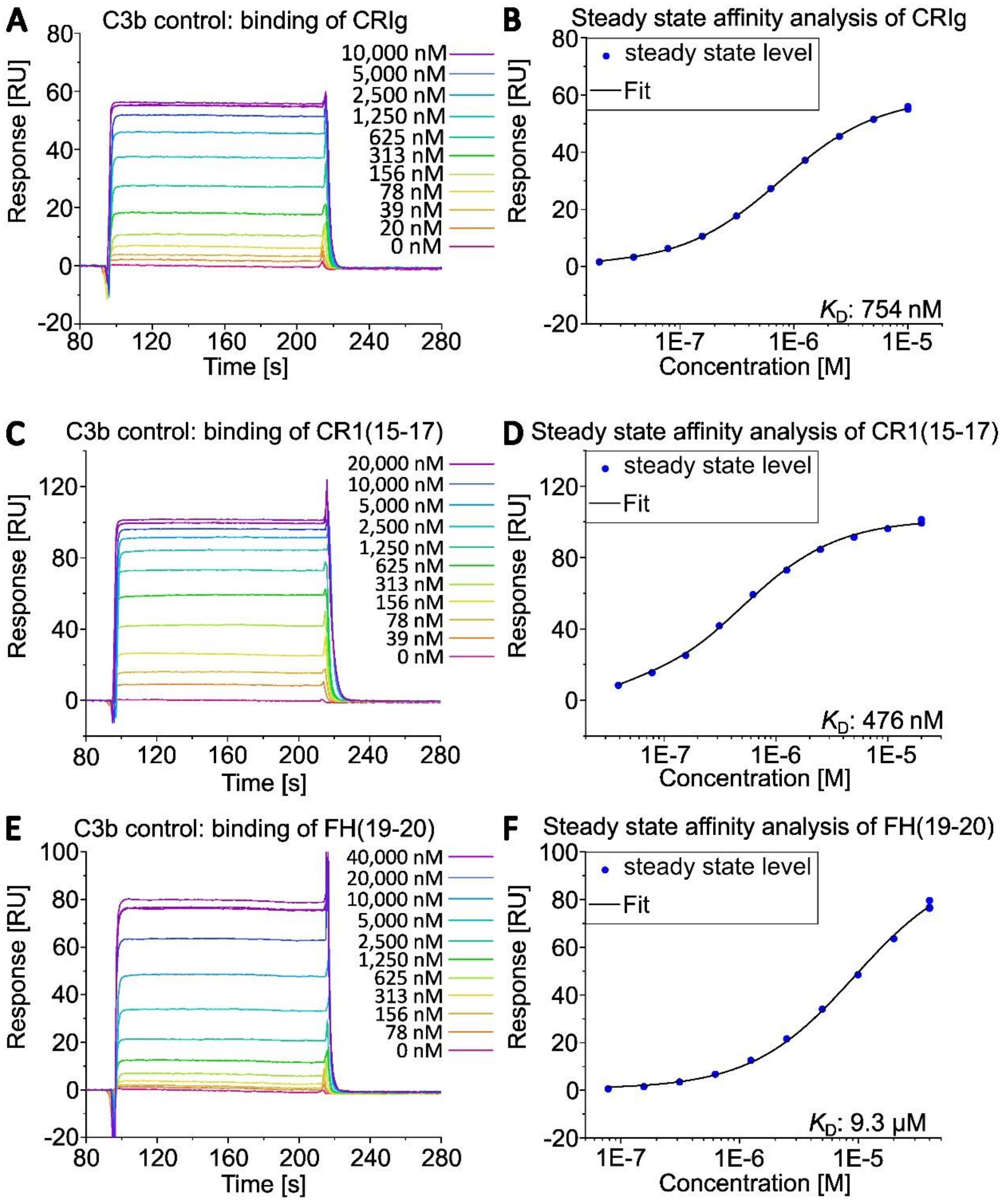

Supplemental Figure 7. Interaction of commercial C3b with different complement regulators assessed by SPR. Commercial C3b was specifically biotinylated at the sulfhydryl of the thioester moiety and immobilised on a streptavidin (SA) sensorchip (Cytiva). The binding of **A)** CRIg, **C)** CR1(15-17), **E)** FH(19-20) was measured. Steady state affinity fits returned the equilibrium dissociation constants by applying a 1:1 binding model (**B, D, F**) in the Biacore X100 evaluation software.

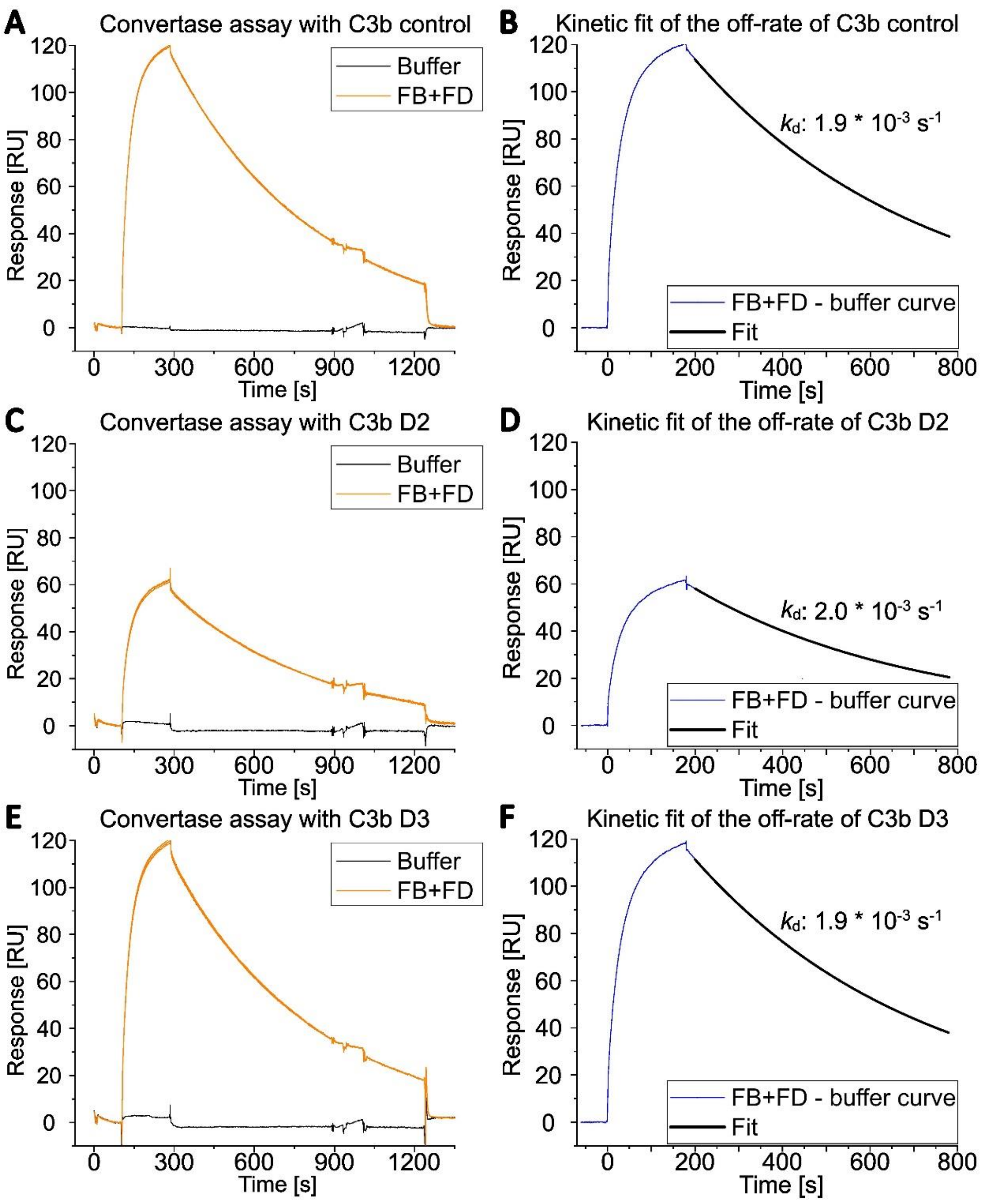

Supplemental Figure 8. Overlay of SPR analysis of convertase assembly and dissociation. Convertase formation by SPR was measured by simultaneously injecting 600 nM FB and 100 nM FD for 3 minutes onto C3b from **A)** a commercial source, **C)** D2 and **E)** D3 immobilised via the thioester moiety (biotinylated sulfhydryl). Regeneration of the surface was achieved by applying CR1(1-3) at 1 µM followed by an injection of 1 M NaCl (not shown). The FB and FD injections were performed in total three times and are shown as overlay of three curves. **B)**, **D)**, **F)** Only one of three curves from *A), C)* and *E)* and its corresponded curve fit is shown after the buffer curve had been subtracted (injection start of FB and FD was set to 0 seconds). The natural convertase dissocation rate (k_d) was estimated by applying an exponential decay fit with OriginPro® evaluation software between 200 to 780 s.

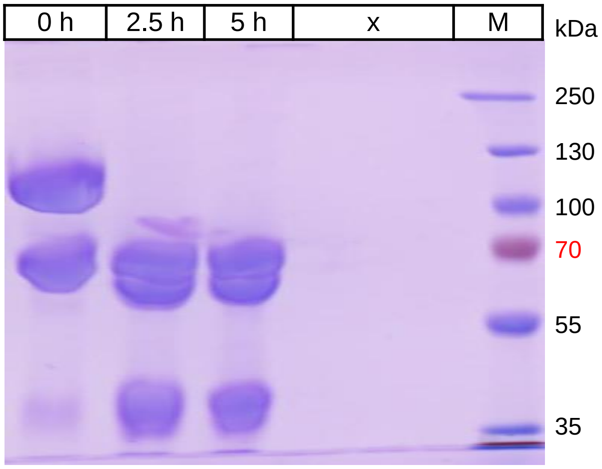

Supplemental Figure 9. Fluid-phase FI susceptibility assay of freshly purified C3b. C3b, that had not been frozen, was incubated with 100 nM FI and 1 µM FH(1-6) at 37 °C for up to 5 h. Samples were reduced with 2-mercaptoethanol, heat-inactivated and approx. 9 µg of C3b were applied to each well of the 9 % SDS-PAGE gel. Molecular weight marker as indicated.

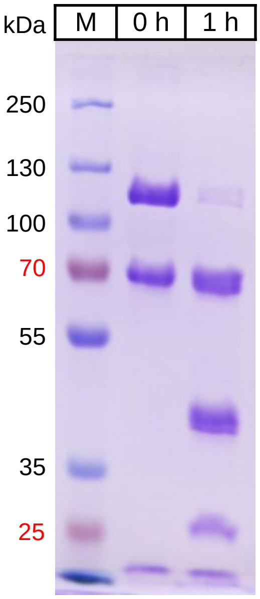

Supplemental Figure 10. Fluid-phase Factor I functional assay of commercial C3b (thawed only once) with Factor I obtained from the FI-depletion step. Commercial C3b was incubated with 1 µM CR1(15-17) and approx. 100 nM FI of one donor at 37 °C. Samples were reduced with 2-mercaptoethanol, heat-inactivated and approx. 2 µg of C3b were applied to each well of the 9 % SDS-PAGE gel. Molecular weight marker as indicated.
